# Supplementary material for: The interactome of intact mitochondria by cross-linking mass spectrometry provides evidence for coexisting respiratory supercomplexes
Source: Mol Cell Proteomics. 2017 Dec 8;17(2):216–32. doi: 10.1074/mcp.RA117.000470 (PMC5795388; doi:10.1074/mcp.RA117.000470)
Supplement: Supplemental Data [file supp_RA117.000470_133922_0_supp_23978_fzffwf.zip › spectra_annotation/mito_spectra_annotation/375-1-8-1-2-1.pdf]

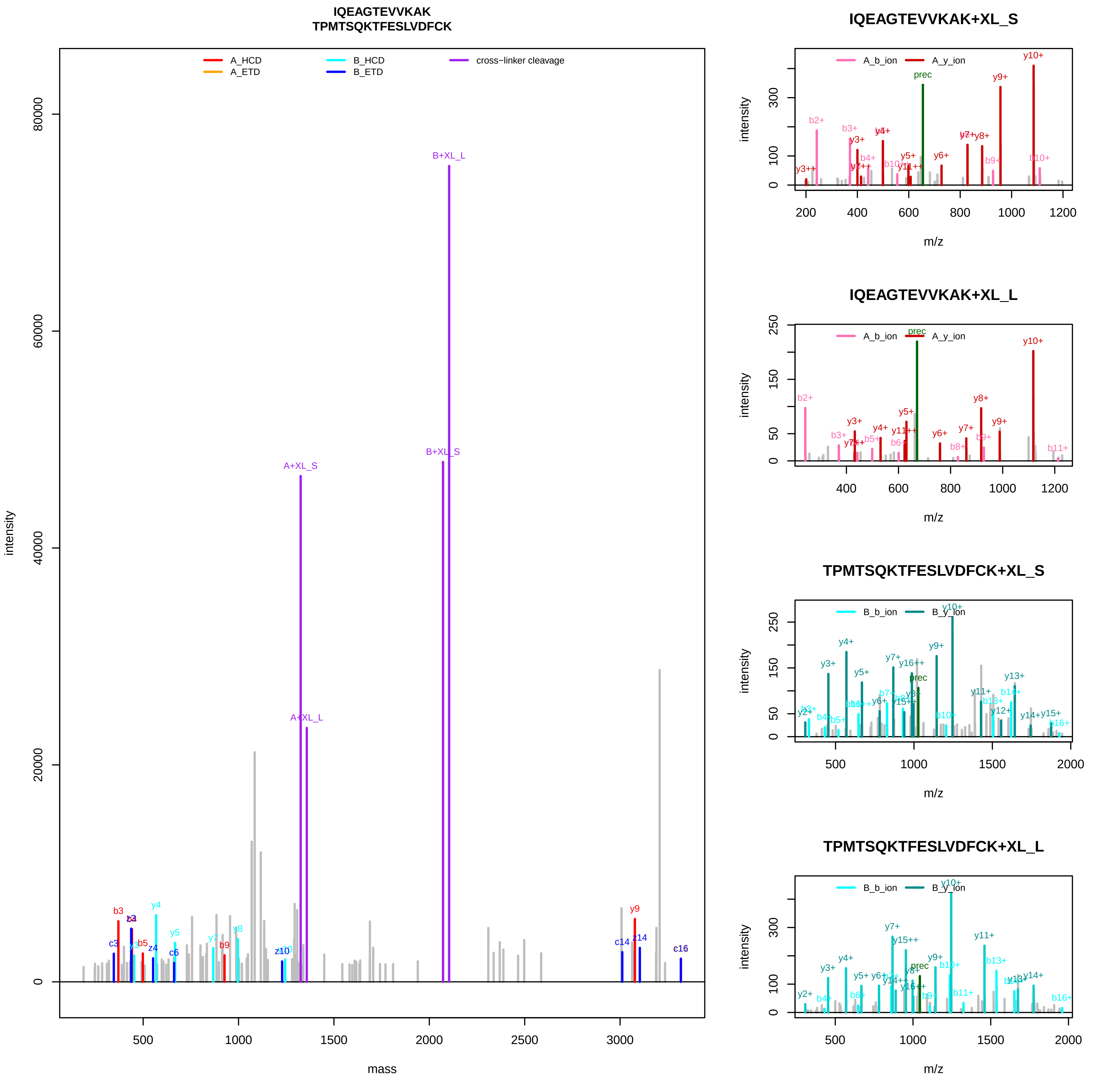

IQEAGTEVVKAK
TPMTSQKTFESLVDFCK
A_HCD
A_ETD
B_HCD
B_ETD
cross−linker cleavage
B+XL_L
B+XL_S
A+XL_S
A+XL_L
b3
y4
c3
y3
b5
z4
c6
y5
y7
b9
y8
z10
c14
y9
z14
c16
intensity
mass
0
20000
40000
60000
80000
500
1000
1500
2000
2500
3000
IQEAGTEVVKAK+XL_S
A_b_ion
A_y_ion
prec
y10+
y9+
b2+
b3+
y4+
y3+
y7+
y8+
b4+
y5+
y6+
y3++
b10+
b9+
m/z
IQEAGTEVVKAK+XL_L
b2+
y10+
y8+
y5+
y3+
y4+
y11++
y6+
y7+
y9+
b3+
b5+
b6+
b8+
b11+
TPMTSQKTFESLVDFCK+XL_S
B_b_ion
B_y_ion
y10+
y4+
y9+
y7+
y16++
y3+
y5+
y13+
y11+
b14+
b13+
y12+
y2+
b3+
b4+
b5+
y6+
y15++
b10+
y14+
y15+
b16+
TPMTSQKTFESLVDFCK+XL_L
y7+
y15++
y11+
y4+
y3+
y9+
b10+
b13+
y5+
y6+
y14++
y8+
y16++
y13+
y14+
y2+
b4+
b6+
b9+
b11+
b16+
